# Microbial community and fermentation characteristic of whole-crop wheat silage treated by lactic acid bacteria and *Artemisia argyi* during ensiling and aerobic exposure

**DOI:** 10.3389/fmicb.2022.1004495

**Published:** 2022-11-10

**Authors:** Zhenyu Wang, Zhongfang Tan, Guofang Wu, Lei Wang, Guangyong Qin, Yanping Wang, Huili Pang

**Affiliations:** ^1^Henan Key Lab Ion Beam Bioengineering, School of Agricultural Sciences, Zhengzhou University, Zhengzhou, China; ^2^Plateau Livestock Genetic Resources Protection and Innovative Utilization Key Laboratory of Qinghai Province, Key Laboratory of Animal Genetics and Breeding on Tibetan Plateau, Ministry of Agriculture and Rural Affairs, Qinghai Academy of Animal and Veterinary Medicine, Qinghai University, Xining, China

**Keywords:** whole crop wheat silage, *Artemisia argyi*, fermentation characteristic, microbial community, mycotoxin

## Abstract

Whole-crop wheat silage (WCWS) is an excellent feed material for ruminants. However, microbial fermentation during silage production consumes valuable nutrients, decreasing the quality of silage. The main objective of this study was to assess how the addition of increasing amounts of *Artemisia argyi* (AA) affected fermentation quality, microbial composition, and mycotoxin production in whole-crop wheat at dough stage (WCWD) silage during ensiling to aerobic exposure compared with *Lactiplantibacillus buchneri* (LB). The addition of 20% AA, resulted in a lower pH and higher lactic acid content, was found in silage treated with 20% AA, and an obvious increase in the relative abundance of *Lactobacillus* was detected in silages treated with LB and 20% AA, respectively. Meanwhile, inoculation with 20% AA decreased the abundance of harmful microorganisms, including *Acinetobacter*, *Enterobacter*, and *Aspergillus*. It also reduced the contents of mycotoxins, Aflatoxin B1 (AFB1), and deoxynivalenol (DON) during ensiling and aerobic exposure. These results confirmed that WCWD treated with 20% AA could improve the fermentation quality and enhance the aerobic stability of silage.

## Introduction

Due to the impact of COVID-19, climatic change, war, and other factors, the prices of bulk raw materials such as corn and soybean meal (SBM) have risen sharply, squeezing the profit margins of breeders. To control costs alternative forms of animal feed need to be urgently identified. Wheat (*Triticum aestivum* L.) is a versatile crop that is used for grain, pasture, or silage in worldwide (Shaani et al., 2017). It presents a high nutritive value, protein content, productivity, and digestibility and also contains good vitamins concentrations and fiber digestibility or even significantly higher than hay and corn (Crovetto et al., 1998). As a result, some herbivores absorb nutrients from wheat better than from high-quality alfalfa (Filya, 2003). An additional advantage of wheat is that it can be grown in the winter in semi-arid climates with yields approaching about 10 tons of dry matter (DM) per hectare (Ashbell et al., 1997). Ensiling is an important method for the preservation of fresh forage. It can reduce environmental risks and supply livestock with nutritious and palatable feedstuff throughout the year (Ren et al., 2021). Whole-crop wheat silage (WCWS) is a good feed material for ruminants, providing fiber and energy for ruminants. When the supply of whole-crop corn silage (WCCS) is insufficient, feeding WCWS can improve the milk yield of dairy cows (Bal and Bal, 2012); therefore, WCWS is widely used in dairy production in developed countries. However, during silage production microbial fermentation will consume a significant proportion of nutrients, reducing the quality of silage. Unfortunately, WCWS follows the same tendency (Ni et al., 2015). Moreover, due to the temperature and relative humidity rise under aerobic condition after silage opening, it is inevitable to proliferate yeast, mold, and other undesirable microorganisms. The aerobic deterioration results in a loss of nutrients up to 30% DM, and mycotoxins production (Borreani et al., 2018; Ferrero et al., 2019). Therefore, in order to improve the nutritional value of WCWS, additives are often used in the production process (Ni et al., 2015).

Both homo- and hetero-fermentative varieties of lactic acid bacteria (LAB) are often used as additives to improve fermentation quality and aerobic stability (Silva et al., 2021). The application of hetero-LAB is more common, especially *Lactiplantibacillus* (*L.*) *buchneri*. However, the effects of additives on the fermentation quality are inconsistent (Filya et al., 2004; Kleinschmit and Kung, 2006). Thus, it is important to develop new additives that can effectively control aerobic stability and inhibit secondary fermentation to ensure silage quality. *Artemisia argyi* H. Lev. & *Vaniot* (AA), a pure natural, green and edible wild herb, is widely distributed in China and east Asia, and has potential nutritional, medicinal, and commercial value (Song et al., 2019). It contains varieties active components, such as essential oils, polysaccharides, flavonoids, phenols, and terpenoids glycosides, which have certain inhibitory effects on mycotoxins Aflatoxin B1 (AFB1) and deoxynivalenol (DON). Additionally, AA is rich in crude protein (CP), polyunsaturated fatty acids, vitamin C, and essential amino acids (Khalaji et al., 2011). Recently, it also gained increasing use as a feed additive in animal husbandry for decreasing diarrhea rate and index and to regulate gastrointestinal tract function in animals (Kim et al., 2012). It is also used to increase serum antioxidant capacity (Zhang et al., 2020), improve fur color or quality and the breeding environment (Popović et al., 2017). In conclusion, the use of AA or its bioactive substance, as a feed additive not only improves animal yield but also reduces feed cost and effectively decreases the probability of secondary fermentation.

The nutritive value of wheat silage varies according to cultivar and stage of maturity at harvest (Ashbell et al., 1997). Wheat at dough stage had relatively low fermentation losses and was quite stable under aerobic exposure (Xia et al., 2018). Thus, the main objective of this work was to assess the effects of AA with different proportions addition on fermentation quality, microbial composition, mycotoxin production, and aerobic stability of whole-crop wheat at dough stage (WCWD) silage by compared with *L. buchneri*. It can provide a theoretical basis for further study on the effects of AA addition on aerobic stability of wheat silage at different stages.

## Materials and methods

### Materials collection and silage preparation

In June 2021, WCWD (cultivated variety, Qiule 18) and whole-crop AA (cultivated variety, Tangyin Beiai) were harvested in Zhengzhou (34.37° N, 113.28° E) and Anyang, Henan Province, China (35.92° N, 114.35° E), respectively. Both of them were chopped into approximately 1–2 cm length for use. *L. buchneri* was donated by China Agricultural University. Single colonies of *L. buchneri* was cultured in Man Rogosa Sharpe (MRS) medium at 37°C for 12 h, then, centrifuged the culture at 12,000 *g* for 10 min at 4°C and mixed precipitate with distilled water to make OD_600_ to 0.80.

Experimental treatments were designed as seven treatments based on the fresh weight including: (1) WCWD, no additive; (2) LB, WCWD with 2% *L. buchneri* [1.00 × 10^6^ colony-forming unit per milliliter (cfu/mL)]; (3) 20% AA (WCWD with 20% AA, next same); (4) 40% AA; (5) 60% AA; (6) 80% AA; and (7) AA (only whole-crop AA). The moisture level of each group was all adjusted to approximately 65%. Forage mixtures (300 g) were placed into special fermentation plastic bag (25 cm × 35 cm) and sealed with a vacuum sealer (P-290, Shineye, Dongguan, China). Each treatment was prepared in quadruplicate and fermented for 1, 3, 7, 15, 30, and 60 days, then aerobic exposure for 2, 4, and 12 days under room temperature (16–35°C). After each opening, samples were taken for microbiological, fermentation quality, chemical composition, and toxin content analysis.

### Fermentation quality, chemical composition, and microbial population analyses

A sample of 10 g was combined with 90 ml of distilled water and then filtered to determine pH value and ammonia nitrogen (NH_3_-N) and was measured using pH meter (Mettler Toledo Co., Ltd., Greifensee, Switzerland) and phenol–hypochloric acid colorimetric method provided by Broderick and Kang (1980), respectively. A high-performance liquid chromatography (HPLC) method was used to measure organic acids according to Wang et al. (2022a).

The sample was dried at 65°C for 48 h to determine DM level and ground through a 1.0-mm sieve to be used for determination of water soluble carbohydrates (WSC), neutral detergent fiber (NDF), acid detergent fiber (ADF), and CP. The WSC was done in accordance with anthrone colorimetry using spectrophotometer (UV mini-1240, Shimadzu, Tokyo, Japan) (Thomas, 1977), NDF and ADF contents were analyzed according to Van Soest et al. (1991), and CP was determined by the Kjeldahl apparatus (K9860, Hainon, Shandong, China) following the procedure of Association of Official Analytical Chemists (AOAC, 1990; method 984.13).

For microbial population analyses, 10 g silage sample was homogenized with 90 mL sterilized normal saline, and then serially diluted. According to the procedure described by Pang et al. (2011), the numbers of LAB were measured by plate count on de MRS agar incubated at 37°C for 48 h under an anaerobic incubator, Potato Dextrose Aga (PDA, containing 0.15% of tartaric acid) incubated at 37°C for 48 h to enumerate yeast, Nutrient Agar (NA) at 37°C for 48 h to enumerate bacillus and aerobic bacteria, respectively. For bacillus, the sample needed to be treated in 75°C water bath for 15 min first. The colonies were counted as the numbers of viable microorganisms in cfu/g of fresh matter (FM).

By comparing the differences in fermentation quality, chemical composition, and microbial population among groups in each period, AA treated group with the relatively best advantages would be selected for further analysis and comparison.

### Toxin contents determination

Enzyme-linked immunosorbent assay (ELISA) kits provided by Lianshuo Biological Technology Co., Ltd (AMEKO, Shanghai, China) were used to analyze mycotoxins including Aflatoxin B1 (AFB1) and deoxynivalenol (DON).

### Bacterial and fungi community analyses

A 10 g frozen sample was placed in 40-mL sterile water, and after homogenization, filtered with two layers of sterile medical gauze, then the gauze was rinsed with 40 mL sterile water three times to recover the residual microorganisms, which were recycled using a centrifuge of 12,000 *g* for 15 min at 4°C after the filtrate was combined. The DNA extraction and polymerase chain reaction amplification were operated according to the method described by Zhou et al. (2021). The DNA samples were paired-end sequenced through an Illumina MiSeq PE300 platform (Majorbio Bio-Pharm Technology Co., Ltd., Shanghai, China).

The sequencing data were analyzed on the Majorbio Bio-Pharm cloud platform.^1^ Community structure was analyzed at the phylum and genus levels using the Silva database with a confidence threshold of 90%. Meanwhile, alpha diversity on OTU level was applied in analyzing the complexity of species diversity for a sample through Chao and Shannon, and principal co-ordinates analysis was demonstrated for the variance of the bacterial and fungi community structure. Spearman’s correlation heatmap analysis was conducted to explore the relationships between microbial community and fermentation products.

### Statistical analyses

The IBM Statistical Package for the Social Sciences statistical package 22.0 (SPSS 22.0. SPSS Inc., Chicago, IL, USA) was used to analyzed the data of fermentation quality, chemical composition, and microbial population, and Duncan tests were used to evaluate differences among treatments. Differences were considered significant when *p* < 0.05.

## Results

### Fermentation quality, chemical composition, and microbial population of fresh materials and silage

Table 1 showed the characteristics of fresh materials. The pH of WCWD and AA were both above 6.00. The contents of NDF and ADF in WCWD were 575.00 and 315.00 g/kg DM, while in AA were 490.00 and 350.00 g/kg DM, respectively. In addition, the counts of epiphytic microorganisms and mycotoxins contents were similar, LAB in two materials were both above 5.50 lg cfu/g FM, the AFB1 and DON levels were below 3.8 μg/kg and 0.15 mg/kg, respectively.

**TABLE 1:** Fermentation quality, chemical composition, and microbial population of fresh materials.

Item		WCWD	AA
DM (%)		44.48	29.50
Fermentation characteristic (g/kg DM)	pH	6.17	6.24
	NH_3_-N	7.13	9.96
	Lactic acid	2.16	1.35
Chemical composition (g/kg DM)	WSC	34.3	59.3
	NDF	575.00	490.00
	ADF	315.00	350.00
	CP	88.23	179.66
Microbial population (lg cfu/g of FM)	LAB	5.64	5.57
	Bacillus	7.89	7.74
	Aerobic bacteria	9.48	8.03
	Yeast	4.45	4.36
Mycotoxin	AFB1, μg/kg	3.59	3.84
	DON, mg/kg	0.11	0.13

According to Table 2, after 3 days ensiling, pH in LB and 20% AA were first lowered than 4.00, NH_3_-N were significantly lower than other groups and lactic acid contents were dramatically higher in these two groups. Lactic acid reached the highest level at 15 days of fermentation in all groups, among which WCWD was the lowest and kept until the end of fermentation. During the aerobic phase, all treated groups had lower pH values and higher lactic acid contents, in particular LB and 20% AA.

**TABLE 2:** Dynamics changes of DM and fermentation quality through silage ensiling.

Item	Treatment	Days of ensiling (day)						Days of aerobic exposure (day)			SEM	*P* value		
		1	3	7	15	30	60	2	4	12		T	E	T × E
DM (%)	WCWD	42.38 ^aB^	42.60 ^aC^	46.43 ^aB^	45.00 ^aB^	43.27 ^aB^	40.12 ^aC^	43.98 ^aB^	51.28 ^aB^	81.82 ^aA^	2.58	<0.01	<0.01	<0.01
	LB	44.01 ^aC^	43.13 ^aC^	41.76 ^aC^	41.66 ^aC^	42.12 ^aC^	45.70 ^aB^	48.40 ^aB^	51.32 ^aB^	75.03 ^aA^				
	20% AA	39.86 ^aB^	42.53 ^aB^	40.09 ^aB^	39.51 ^aB^	42.10 ^aB^	42.46 ^aB^	43.23 ^aB^	44.36 ^bB^	62.51 ^bA^				
	40%AA	38.01 ^aB^	39.62 ^aB^	33.29 ^bB^	34.22 ^bB^	39.45 ^aB^	36.00 ^bB^	39.20 ^bB^	41.54 ^bB^	49.43 ^cA^				
	60% AA	33.86 ^bB^	35.91 ^bB^	32.32 ^adbB^	34.43 ^bB^	33.18 ^bB^	32.74 ^bB^	36.55 ^bB^	39.67 ^bA^	44.28 ^cA^				
	80% AA	33.22 ^bB^	32.85 ^bB^	28.43 ^bB^	28.93 ^bB^	29.85 ^bB^	31.48 ^bB^	31.77 ^bB^	35.01 ^cA^	40.30 ^dA^				
	AA	33.37 ^bA^	28.03 ^bB^	26.42 ^bB^	25.39 ^bB^	26.29 ^bB^	28.26 ^cB^	29.45 ^cB^	29.05 ^cB^	41.71 ^dA^				
pH	WCWD	6.11 ^aA^	4.89 ^aA^	5.74 ^aA^	5.37 ^aA^	5.09 ^aA^	4.74 ^aA^	5.02 ^aA^	5.04 ^aA^	5.87 ^bA^	0.16	<0.05	<0.01	<0.01
	LB	4.25 ^bB^	3.75 ^cC^	3.84 ^cC^	3.84 ^dC^	3.95 ^eB^	3.85 ^cC^	3.77 ^eC^	4.36 ^bB^	6.25 ^aA^				
	20% AA	5.74 ^aA^	3.98 ^cC^	4.22 ^cC^	4.01 ^dC^	4.45 ^cC^	4.11 ^bC^	3.99 ^dC^	4.30 ^cC^	5.32 ^bA^				
	40% AA	5.76 ^aA^	4.01 ^bB^	4.76 ^bB^	4.37 ^cB^	4.40 ^dB^	4.12 ^bB^	4.04 ^dB^	4.21 ^dB^	5.79 ^bA^				
	60% AA	5.97 ^aA^	4.98 ^aB^	5.39 ^aA^	4.73 ^bB^	4.52 ^bC^	4.54 ^aC^	4.15 ^cC^	4.24 ^dC^	5.56 ^cA^				
	80% AA	6.19 ^aA^	5.00 ^aB^	5.48 ^aB^	5.19 ^aB^	4.64 ^bC^	4.41 ^aC^	4.38 ^bC^	4.46 ^bC^	6.04 ^bA^				
	AA	6.21 ^aB^	5.14 ^aC^	5.46 ^aC^	4.81 ^bD^	4.69 ^bD^	4.50 ^aD^	4.42 ^bE^	4.61 ^bD^	6.60 ^aA^				
NH_3_-N (g/kg DM)	WCWD	9.45 ^aA^	10.16 ^bA^	10.52 ^bA^	12.97 ^bA^	12.93 ^bA^	15.09 ^cA^	19.87 ^cA^	13.15 ^bA^	9.52 ^cA^	1.94	<0.01	<0.01	<0.01
	LB	7.57 ^aB^	8.48 ^bB^	9.21 ^bB^	10.67 ^cB^	10.77 ^cB^	12.28^ dB^	11.28^ dB^	11.06 ^cB^	21.95 ^bA^				
	20% AA	9.15 ^aB^	9.82 ^bB^	11.65 ^bB^	14.68 ^bA^	13.70 ^bB^	19.01 ^cA^	17.14 ^cA^	16.17 ^bA^	18.56 ^bA^				
	40% AA	9.41 ^aB^	11.88 ^aB^	14.87 ^aB^	19.40 ^bA^	15.46 ^bB^	22.05 ^bA^	20.30 ^cA^	17.51 ^bA^	15.37 ^bB^				
	60% AA	10.45 ^aC^	13.41 ^aC^	13.62 ^bC^	20.00 ^aB^	18.78 ^bB^	27.87 ^bA^	25.48 ^bA^	21.69 ^aA^	25.33 ^aA^				
	80% AA	10.79 ^aC^	15.88 ^aB^	18.98 ^aB^	25.82 ^aA^	26.83 ^aA^	25.30 ^bA^	38.78 ^aA^	25.16 ^aA^	29.83 ^aA^				
	AA	10.61 ^aC^	17.27 ^aB^	22.20 ^aB^	24.19 ^aB^	26.74 ^aA^	34.06 ^aA^	32.63 ^aA^	24.79 ^aB^	20.82 ^bB^				
Lactic acid (g/kg DM)	WCWD	1.55 ^aA^	2.67 ^aA^	2.47 ^bA^	2.61 ^aA^	3.17 ^aA^	5.78 ^bA^	9.94 ^aA^	3.29 ^aA^	1.63 ^aA^	3.51	<0.01	<0.01	<0.05
	LB	6.17 ^aA^	10.86 ^bA^	10.15 ^aA^	13.56 ^aA^	8.94 ^aA^	10.55 ^bA^	14.73 ^aA^	7.94 ^aA^	7.28 ^aA^				
	20% AA	2.11 ^aA^	7.59 ^bA^	7.72 ^aA^	10.35 ^aA^	4.73 ^aA^	12.14 ^bA^	13.55 ^aA^	9.79 ^aA^	8.36 ^aA^				
	40% AA	1.97 ^aB^	6.03 ^aB^	4.43 ^bB^	10.55 ^aB^	5.37 ^aB^	28.02 ^aA^	13.45 ^aB^	12.63 ^aB^	5.08 ^aB^				
	60% AA	1.56 ^aB^	2.83 ^aB^	3.30 ^bA^	5.16 ^aA^	7.00 ^aA^	14.72 ^bA^	17.48 ^aA^	12.51 ^aA^	4.90 ^aA^				
	80% AA	1.59 ^aA^	3.29 ^aA^	3.65 ^bA^	7.29 ^aA^	5.25 ^aA^	8.56 ^bA^	17.65 ^aA^	6.87 ^aA^	5.91 ^aA^				
	AA	1.70 ^aB^	3.47 ^aB^	1.83 ^bB^	9.68 ^aA^	7.37 ^aA^	15.89 ^bA^	10.24 ^aA^	5.23 ^aA^	6.07 ^aA^				
Acetic acid (g/kg DM)	WCWD	1.36 ^aA^	1.76 ^aA^	1.67 ^aA^	2.21 ^bA^	1.73 ^aA^	2.19 ^cA^	3.15 ^aA^	1.29 ^aA^	1.55 ^aA^	1.26	<0.01	<0.01	<0.01
	LB	1.29 ^aA^	1.43 ^aA^	1.58 ^aA^	2.11 ^bA^	1.76 ^aA^	2.48 ^cA^	1.29 ^bA^	1.29 ^aA^	2.32 ^aA^				
	20% AA	1.38 ^aA^	1.72 ^aA^	1.67 ^aA^	2.35 ^bA^	2.17 ^aA^	4.46 ^bA^	3.12 ^aA^	1.29 ^aA^	1.29 ^aA^				
	40%AA	1.38 ^aB^	1.77 ^aB^	1.60 ^aB^	3.35 ^aB^	2.53 ^aB^	11.59 ^aA^	2.48 ^aB^	1.29 ^aB^	2.60 ^aB^				
	60% AA	1.29 ^aB^	1.81 ^aB^	1.64 ^aB^	2.99 ^aA^	1.99 ^aB^	6.91 ^bA^	3.22 ^aA^	2.23 ^aB^	1.29 ^aB^				
	80% AA	1.29 ^aB^	1.94 ^aB^	2.49 ^aB^	2.41 ^aB^	2.97 ^aB^	11.07 ^aA^	4.32 ^aB^	1.94 ^aB^	1.81 ^aB^				
	AA	1.39 ^aB^	2.22 ^aB^	2.39 ^aA^	6.29 ^aA^	3.87 ^aA^	6.42 ^bA^	2.83 ^aA^	1.69 ^aB^	1.64 ^aB^				

NH_3_-N, ammonia nitrogen; DM, dry matter. WCWD, no additive; LB, WCWD with 2% *L. buchneri*; 20% AA, WCWD with 20% AA, next same; 40% AA; 60% AA; 80% AA; AA, only whole-crop AA. Means with different letters in the same row (A–D) or column (a–e) indicate a significant difference according to Duncan test (*p* < 0.05). SEM, standard error of means; T, treatment; E, ensiling days; T × E, interaction between treatment and ensiling days.

Chemical compositions of silage are shown in Table 3. The WSC and CP were relatively higher in all treated groups except LB on 3 days, and the contents increased as AA proportion was added. Moreover, NDF content in all AA groups was always lower than that in WCWD and LB. At 2 days of aerobic exposure, DM contents were increased in all groups. With the aerobic exposure time prolonged, AA group had the highest WSC content, and the second was the 20% AA group.

**TABLE 3:** Chemical composition through silage ensiling and aerobic exposure.

Item	Treatment	Days of ensiling (day)						Days of aerobic exposure (day)			SEM	*P* value		
		1	3	7	15	30	60	2	4	12		T	E	T × E
WSC (g/kg DM)	WCWD	52.13 ^cA^	39.42^ dB^	31.92 ^eC^	29.38 ^dC^	28.63 ^eD^	26.18 ^dD^	24.30 ^dD^	21.49 ^dF^	10.58 ^dF^	0.82	<0.01	<0.05	<0.01
	LB	54.59 ^cA^	38.58^ dB^	33.43 ^eC^	31.21 ^dC^	29.90 ^eD^	27.47 ^dD^	26.22 ^dD^	25.65 ^bD^	20.21 ^cE^				
	20% AA	55.19 ^cA^	50.16 ^cB^	39.79 ^dC^	37.12 ^cD^	34.66 ^dD^	31.32 ^cE^	29.14 ^bF^	27.87 ^bF^	24.64 ^bF^				
	40% AA	58.50 ^bA^	52.34 ^bB^	47.06 ^cC^	42.27 ^bD^	32.27 ^dE^	30.70 ^cE^	27.90 ^cF^	27.07 ^bF^	21.62 ^bF^				
	60% AA	60.40 ^bA^	40.50^ dB^	57.87 ^aA^	41.54 ^bB^	38.27 ^cC^	34.53 ^bD^	31.06 ^bE^	26.89 ^bE^	19.75 ^cF^				
	80% AA	63.50 ^aA^	58.25 ^aB^	51.45 ^bC^	49.06 ^aC^	40.97 ^bD^	30.27 ^cE^	28.25 ^cE^	24.24 ^cF^	18.77 ^cF^				
	AA	64.10 ^aA^	59.30 ^aB^	53.87 ^bC^	48.01 ^aD^	44.12 ^aE^	40.50 ^aE^	38.76 ^aE^	36.20 ^aF^	30.40 ^aF^				
NDF (g/kg DM)	WCWD	565.00 ^aA^	554.45 ^aB^	541.58 ^aC^	534.43 ^aC^	524.63 ^aD^	518.69 ^aE^	512.28 ^aE^	497.75 ^aF^	479.15 ^aF^	0.81	<0.01	<0.01	<0.01
	LB	558.76 ^bA^	548.28 ^aA^	532.03 ^bB^	524.61 ^bC^	521.29 ^aC^	507.10 ^bD^	500.00 ^bD^	492.27 ^bE^	470.87 ^bF^				
	20% AA	549.27 ^bA^	533.94 ^bB^	523.68 ^cC^	519.61 ^bD^	517.00 ^bD^	502.65 ^bE^	496.06 ^bF^	481.79 ^cF^	465.17 ^cF^				
	40% AA	539.22 ^cA^	526.29 ^bB^	519.71^ dB^	509.21 ^cC^	502.57 ^cC^	497.43 ^bD^	493.12 ^bD^	487.70 ^bE^	460.12 ^dF^				
	60% AA	528.67 ^dA^	514.29 ^Bc^	509.26^ dB^	500.55 ^cC^	495.00 ^cC^	485.94 ^cD^	481.59 ^cD^	471.11 ^dE^	463.45 ^cF^				
	80% AA	507.14 ^eA^	500.18^ dB^	498.12 ^eB^	495.10 ^dC^	483.94 ^dD^	478.84 ^dD^	475.24 ^cD^	464.75 ^dE^	459.21 ^dE^				
	AA	483.42 ^eA^	480.20^ dB^	474.55 ^eB^	471.43 ^eB^	467.05 ^eC^	460.77 ^eC^	455.14 ^dD^	448.29 ^eD^	432.38 ^eE^				
ADF (g/kg DM)	WCWD	310.00 ^eA^	304.79 ^cB^	303.24^ dB^	298.51 ^eC^	294.39 ^dC^	290.46 ^dD^	287.86 ^eE^	286.39 ^dE^	284.45 ^eE^	0.82	<0.01	<0.01	<0.01
	LB	305.83 ^eA^	300.72 ^cB^	299.89 ^eB^	294.72 ^eC^	288.42 ^dD^	286.79 ^eD^	282.94 ^eE^	279.15 ^eE^	274.27 ^eF^				
	20% AA	318.42 ^dA^	315.51 ^bB^	313.08 ^cB^	304.45 ^dC^	300.49 ^cC^	294.23 ^dD^	291.72 ^dD^	289.22 ^dE^	283.98 ^eF^				
	40% AA	324.72 ^cA^	322.46 ^aA^	319.43 ^bB^	313.47 ^cC^	309.82 ^cD^	305.85 ^cD^	300.90 ^cE^	298.57 ^cE^	295.08 ^dF^				
	60% AA	330.08 ^bA^	327.00 ^aB^	325.64 ^aB^	322.21 ^bC^	318.89 ^bD^	314.75 ^bE^	312.72 ^bE^	311.25 ^bE^	308.45 ^cF^				
	80% AA	335.26 ^bA^	331.68 ^aB^	328.70 ^aC^	325.80 ^bC^	322.00 ^bD^	320.14 ^aD^	318.02 ^aD^	317.24 ^aD^	314.05 ^bE^				
	AA	342.36 ^aA^	335.90 ^aB^	331.67 ^aC^	329.30 ^aC^	326.08 ^aC^	321.99 ^aD^	319.06 ^aE^	318.28 ^aE^	316.84 ^aE^				
CP (g/kg DM)	WCWD	84.84 ^cA^	88.20 ^cA^	88.77 ^bA^	85.83 ^cA^	88.97 ^cA^	82.17 ^dA^	85.67 ^cA^	93.47 ^bA^	80.09 ^cA^	4.37	<0.05	<0.05	<0.05
	LB	82.61 ^cA^	83.60 ^dA^	82.30 ^cA^	88.37 ^cA^	81.77 ^cA^	87.77 ^dA^	84.14 ^cA^	86.07 ^cA^	81.48 ^bB^				
	20% AA	85.01 ^cB^	82.50^ dB^	86.03 ^cB^	85.80 ^cB^	90.74 ^cB^	144.97 ^bA^	88.67 ^cB^	96.83 ^bB^	86.60 ^bB^				
	40% AA	87.35 ^bB^	81.47^ dB^	87.80 ^cB^	91.08 ^cB^	86.77 ^cB^	107.07 ^cA^	89.93 ^cB^	91.53 ^cB^	88.53 ^bB^				
	60% AA	103.97 ^bA^	98.07 ^cB^	87.47 ^cB^	99.17 ^bA^	90.50 ^cB^	99.14 ^cA^	113.40 ^bA^	105.50 ^bA^	94.52 ^bB^				
	80% AA	158.50 ^aA^	113.77 ^bB^	105.40 ^bB^	115.67 ^bA^	115.27 ^bA^	113.17 ^cB^	128.73 ^aA^	109.23 ^aB^	109.60 ^aB^				
	AA	162.67 ^aA^	135.43 ^aB^	127.61 ^aB^	140.17 ^aB^	133.03 ^aB^	127.63 ^aB^	129.53 ^aB^	121.37 ^aB^	120.04 ^aC^				

WSC, water soluble carbohydrates; NDF, neutral detergent fiber; ADF, acid detergent fiber; CP, crude protein. WCWD, no additive; LB, WCWD with 2% *L. buchneri*; 20% AA, WCWD with 20% AA, next same; 40% AA; 60% AA; 80% AA; AA, only whole-crop AA. Means with different letters in the same row (A–F) or column (a–e) indicate a significant difference according to Duncan test (*p* < 0.05). SEM, standard error of means; T, treatment; E, ensiling days; T × E, interaction between treatment and ensiling days.

With the extension of ensiling, the decrease rate of harmful microorganisms in AA-added groups was significantly higher than WCWD (*p* < 0.01) (Table 4). In LB, the number of LAB was relatively higher. On day 7, the number of LAB in 20% AA began to the highest with 9.96 lg cfu/g FM. During the whole aerobic exposure phase, 20% AA group was still the highest in each stage with above 8.00 lg cfu/g FM on 12 days.

**TABLE 4:** Microbial population through silage ensiling and aerobic exposure.

Item	Treatment	Days of ensiling (day)						Days of aerobic exposure (day)			SEM	*P* value		
		1	3	7	15	30	60	2	4	12		T	E	T × E
Lactic acid bacteria	WCWD	8.10 ^bB^	9.01 ^bA^	9.64 ^aA^	8.99 ^aA^	7.33 ^bB^	7.32 ^bB^	7.70 ^aB^	8.35 ^aA^	8.22 ^aA^	0.19	<0.01	<0.01	<0.01
	LB	10.17 ^aA^	9.78 ^aA^	9.96 ^aA^	9.37 ^aA^	7.49 ^bB^	6.05 ^cC^	7.79 ^aB^	8.91 ^aA^	9.52 ^aA^				
	20% AA	8.03 ^bB^	9.76 ^aA^	9.84 ^aA^	9.49 ^aA^	8.44 ^aB^	7.42 ^bC^	8.02 ^aC^	9.10 ^aA^	9.91 ^aA^				
	40% AA	8.25 ^bB^	9.04 ^bA^	9.74 ^aA^	9.38 ^aA^	8.67 ^aB^	7.56 ^bC^	7.64 ^aB^	9.18 ^aA^	9.80 ^aA^				
	60% AA	7.94 ^bB^	9.73 ^aA^	9.67 ^aA^	9.36 ^aA^	8.98 ^aA^	7.79 ^bB^	5.59 ^bC^	7.27 ^bB^	9.59 ^aA^				
	80% AA	7.84 ^bB^	9.78 ^aA^	9.83 ^aA^	9.20 ^aA^	9.07 ^aA^	8.23 ^aB^	6.59 ^bC^	7.20 ^bB^	9.87 ^aA^				
	AA	8.00 ^bB^	9.92 ^aA^	9.62 ^aA^	9.51 ^aA^	8.85 ^aA^	8.85 ^aA^	7.06 ^aC^	6.59 ^bC^	9.53 ^aA^				
Bacillus	WCWD	9.89 ^aA^	9.45 ^aA^	7.69 ^bB^	7.63 ^aB^	5.61 ^aC^	5.51 ^aC^	7.06 ^aB^	7.09 ^bB^	9.11 ^aA^	0.22	<0.01	<0.01	<0.01
	LB	7.01 ^bB^	3.85 ^cC^	4.05 ^cC^	ND ^cD^	4.58 ^aC^	4.13 ^bC^	7.21 ^aB^	9.14 ^aA^	9.63 ^aA^				
	20% AA	9.73 ^aA^	7.68 ^bB^	7.05 ^bB^	5.63 ^bC^	4.49 ^aD^	3.80 ^bD^	5.67 ^bC^	8.82 ^aA^	8.34 ^aA^				
	40% AA	9.81 ^aA^	8.72 ^aB^	7.46 ^bB^	7.39 ^aC^	4.41 ^aD^	3.70 ^bE^	6.12 ^bD^	5.02 ^cD^	9.24 ^aA^				
	60% AA	9.21 ^aA^	9.11 ^aA^	7.82 ^bB^	7.64 ^aB^	4.69 ^aC^	4.03 ^bD^	3.98 ^cD^	5.10 ^cC^	8.57 ^aA^				
	80% AA	9.20 ^aA^	9.04 ^aA^	9.17 ^aA^	7.64 ^aB^	4.71 ^aC^	3.86 ^bC^	3.90 ^cC^	4.36 ^cC^	9.18 ^aA^				
	AA	8.99 ^aA^	9.65 ^aA^	9.29 ^aA^	7.50 ^aB^	4.89 ^aC^	3.90 ^bD^	3.70 ^cD^	4.46 ^cC^	9.38 ^aA^				
Aerobic bacteria	WCWD	9.66 ^aA^	9.24 ^aA^	7.89 ^bB^	7.76 ^bC^	7.63 ^bC^	6.50 ^aC^	7.60 ^bC^	9.05 ^aA^	9.99 ^aA^	0.24	<0.01	<0.01	<0.01
	LB	7.22 ^bB^	4.10 ^cC^	4.99 ^cC^	5.18 ^cC^	9.30 ^aA^	4.74 ^bC^	7.45 ^bB^	9.44 ^aA^	9.73 ^aA^				
	20% AA	9.52 ^aA^	8.18 ^bB^	7.21 ^bC^	4.65 ^cD^	7.19 ^cC^	4.86 ^bD^	5.73 ^cD^	8.81 ^aA^	9.49 ^aA^				
	40% AA	9.93 ^aA^	9.42 ^aA^	7.74 ^bB^	7.35 ^bC^	4.87 ^dD^	4.33 ^bD^	5.39 ^cD^	7.49 ^bC^	8.97 ^bB^				
	60% AA	9.55 ^aA^	9.13 ^aA^	9.19 ^aA^	7.53 ^bB^	5.06 ^dC^	4.73 ^bC^	5.22 ^cC^	5.57 ^bC^	9.19 ^aA^				
	80% AA	9.69 ^aA^	9.05 ^aA^	7.77 ^bB^	8.89 ^aA^	5.05 ^dC^	4.74 ^bC^	9.02 ^aA^	5.38 ^bC^	9.39 ^aA^				
	AA	9.65 ^aA^	9.47 ^aA^	9.33 ^aA^	4.84 ^cB^	4.87 ^dB^	4.67 ^bC^	5.57 ^cB^	5.08 ^bB^	9.84 ^aA^				
Yeast	WCWD	4.47 ^aC^	ND ^bE^	4.22 ^aC^	4.92 ^aC^	ND ^bE^	ND ^cE^	2.34 ^bD^	5.15 ^bB^	7.52 ^aA^	0.90	<0.01	<0.05	<0.01
	LB	ND ^cD^	4.39 ^aB^	ND ^cD^	4.05 ^aB^	5.39 ^aB^	1.68 ^bC^	7.28 ^aA^	8.83 ^aA^	7.70 ^aA^				
	20% AA	3.94 ^bC^	ND ^bD^	4.57 ^aC^	ND ^bD^	ND ^bD^	ND ^cD^	6.50 ^aB^	8.86 ^aA^	6.85 ^cB^				
	40% AA	ND ^cD^	4.35 ^aC^	4.57 ^aC^	ND ^bD^	ND ^bD^	6.58 ^aB^	6.48 ^aB^	8.66 ^aA^	7.00 ^bB^				
	60% AA	4.06 ^aC^	ND ^bE^	ND ^cE^	ND ^bE^	ND ^bE^	1.49 ^bD^	1.80 ^bD^	6.15 ^bB^	7.05 ^bA^				
	80% AA	2.93 ^bC^	ND ^bD^	ND ^cD^	ND ^bD^	ND ^bD^	ND ^cD^	ND ^cD^	5.25 ^bB^	7.03 ^bA^				
	AA	4.09 ^aB^	ND ^bD^	2.96 ^bC^	ND ^bD^	ND ^bD^	ND ^cD^	ND ^cD^	4.93 ^cB^	6.97 ^cA^				

WCWD, no additive; LB, WCWD with 2% *L. buchneri*; 20% AA, WCWD with 20% AA, next same; 40% AA; 60% AA; 80% AA; AA, only whole-crop AA. Means with different letters in the same row (A-E) or column (a-e) indicate a significant difference according to Duncan test (*p* < 0.05). SEM, standard error of means; T, treatment; E, ensiling days; T × E, interaction between treatment and ensiling days.

Combining the above results, a 20% of AA-treated group had been selected for the following toxin contents and microbial communities analysis with LB group.

### Toxin contents among silage

**FIGURE 1:**
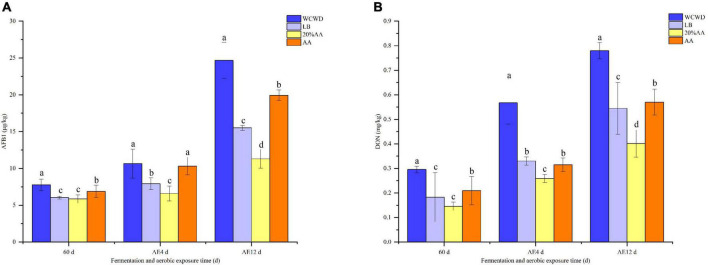
Contents of AFB1 **(A)** and DON **(B)** after 60 days of ensiling and during the aerobic exposure; 60 d, ensiling for 60 days. AE, aerobic exposure; AE4 d and AE12 d, aerobic exposure for 4 and 12 days, respectively. WCWD, no additive; LB, WCWD with 2% *Lactiplantibacillus buchneri*; 20% AA, WCWD with 20% AA; AA, only whole-crop AA. Means with different letters in the same time (a–d) indicate a significant difference according to Duncan test (*p* < 0.05).

Figure 1 exhibited the changes of AFB1 and DON through the ensiling and aerobic exposure. After 60 days fermentation, AFB1 increased in all groups, while in 20% AA still below 5.9 μg/kg; the DON in WCWD was higher than other groups with about 0.30 mg/kg. During the whole aerobic exposure, AFB1 continued to increase, and lowest and highest contents were observed in 20% AA and WCWD in every phase, respectively. Similarly, the DON contents in all groups still increased with the prolongation of aerobic exposure as AFB1.

### Dynamics changes of microbial community on fresh materials and silage

#### Alpha diversity indexes

As shown in Table 5, for bacterial, after 60 days of ensiling, Shannon and Chao1 indexes in all groups were lower than fresh materials. With the aerobic exposure time prolonged, Shannon indexes in WCWD increased, while LB and 20% AA decreased. Chao1 indexes in all groups decreased, especially in 20% AA group. For fungi, Chao1 indexes in all silages after 60 days ensiling were lower than fresh materials. After aerobic exposure of 4 days, Shannon index in WCWD increased; while Chao1 indexes in WCWD, LB and 20% AA decreased, especially in 20% AA.

**TABLE 5:** Alpha diversity of bacteria and fungi during ensiling and aerobic exposure.

Items	Treatments	Bacteria		Fungi	
		Shannon index	Chao1 index	Shannon index	Chao1 index
Fresh materials	WCWD	2.08	339.46	1.28	264.83
	AA	3.02	580.77	2.23	444.71
60 d ensiling	WCWD	1.88	227.48	1.73	96.17
	LB	1.21	338.20	1.78	183.34
	20% AA	1.53	265.77	2.00	262.40
	AA	1.82	292.12	1.86	239.55
aerobic exposure for 4 d	WCWD	2.03	207.52	2.05	41.44
	LB	2.54	264.34	1.26	31.78
	20% AA	2.18	460.43	1.62	52.81
	AA	1.86	307.00	1.17	244.87
aerobic exposure for 12 d	WCWD	3.09	239.85	1.46	46.78
	LB	1.83	139.42	1.62	41.08
	20% AA	1.73	154.30	2.09	41.36
	AA	2.24	162.04	1.16	52.08

WCWD, no additive; LB, WCWD with 2% *L. buchneri*; 20% AA, WCWD with 20% AA; AA, only whole-crop AA.

#### Principal coordinate

**FIGURE 2:**
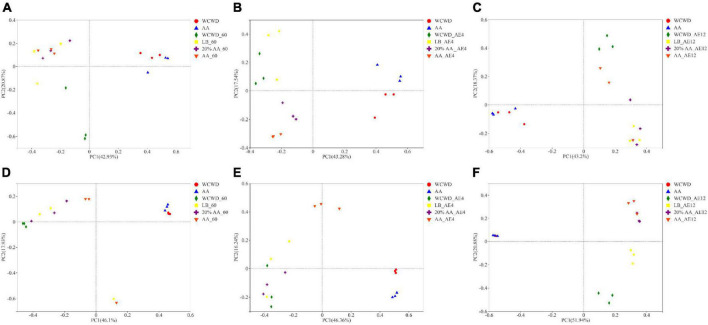
Principal co-ordinates analysis on OTU level of bacterial and fungi community during ensiling and aerobic exposure. **(A,D)** Bacterial and fungi communities after 60 days of ensiling; **(B,C,E,F)**, bacterial and fungi communities after aerobic exposure for 4 and 12 days, respectively. WCWD, no additive; LB, WCWD with 2% *Lactiplantibacillus buchneri*; 20% AA, WCWD with 20% AA; AA, only whole-crop AA.

Microbial communities in all groups were significantly different from that in fresh materials after 60 days (Figure 2). At 12 days of aerobic exposure, bacterial communities of WCWD and AA were more similar, while in LB and 20% AA more analogous. For fungi community, the compositions of fungi in WCWD, LB, and 20% AA were becoming similar on 4 days of aerobic exposure. With the extension of aerobic exposure time, the differences between all groups were gradually obvious; meanwhile, 20% AA and AA were becoming similar.

#### Microbial community abundance

**FIGURE 3:**
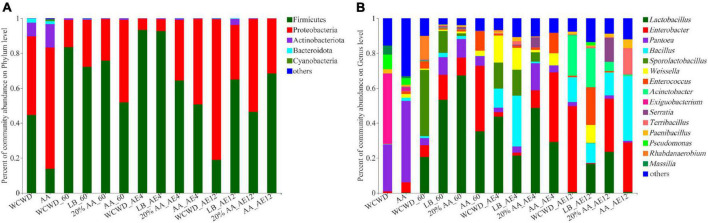
Bacterial community compositions at the levels of phylum **(A)** and genus **(B)**. 60, ensiling for 60 days. AE, aerobic exposure; AE4 and AE12, aerobic exposure for 4 and 12 days, respectively. WCWD, no additive; LB, WCWD with 2% *Lactiplantibacillus buchneri*; 20% AA, WCWD with 20% AA; AA, only whole-crop AA.

As illustrated in Figure 3A, for phylum level, the abundances of Proteobacteria were the dominant community in both two fresh materials, while the predominantly bacterial community shifted from Proteobacteria to Firmicutes after 60 days ensiling. On 12 days aerobic exposure, Proteobacteria regained dominance in WCWD, LB, and 20% AA. As for genus level of bacterial community displayed in Figure 3B, *Lactobacillus* and *Enterobacter* increased and *Pantoea* decreased in WCWD, LB, 20% AA and AA after 60 days. Among aerobic exposure time, the bacterial community structure in all groups comparatively stayed stable, consisting mainly of *Lactobacillus*, *Enterobacter*, *Bacillus*, and *Weissella*. After 12 days aerobic exposure, *Lactobacillus* markedly decreased and *Enterobacter* significantly reduced in WCWD and AA. Meantime, *Lactobacillus* was highest at 16.61%, while *Weissella* (0.08%) and *Acinetobacter* (5.29%) were lowest at 20% AA.

**FIGURE 4:**
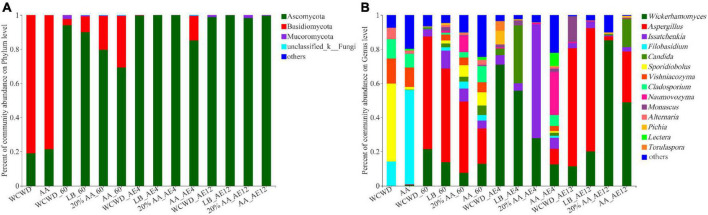
Fungi community compositions at the levels of phylum **(A)** and genus **(B)**. 60, ensiling for 60 days; AE, aerobic exposure, AE4 and AE12, aerobic exposure for 4 and 12 days, respectively. WCWD, no additive; LB, WCWD with 2% *Lactiplantibacillus buchneri*; 20% AA, WCWD with 20% AA; AA, only whole-crop AA.

As can be seen in Figure 4A, the phylum level of fungi communities showed that Ascomycota increased in all silages after 60 days ensiling, among which WCWD had the highest abundance. With the prolongation of aerobic exposure, the abundance of Ascomycota increased, which was higher than Basidiomycota and occupied the dominant position among all groups on 4 and 12 days. For genus level in Figure 4B, *Sporidiobolus* (45.71%) took a dominant position in fresh WCWD and *Filobasidium* (55.34%) took a dominant position in fresh AA. After ensiling 60 days, *Wickerhamomyces* and *Aspergillus* abundances increased, while *Sporidiobolus* and *Filobasidium* decreased. During aerobic exposure, the fungal community composition relatively stayed stable, consisting mainly of *Wickerhamomyces*, *Issatchenkia*, and *Candida*. Compared with other groups, 20% AA had the most *Issatchenkia* abundance (66.69%) and the least *Aspergillus* abundance (1.01%) on day 4.

### Correlation analyses of microbial community and fermentation products

**FIGURE 5:**
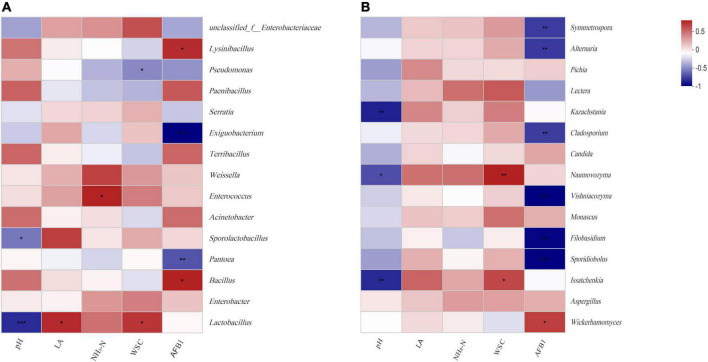
Spearman correlation heatmap of abundance of the top 15 enriched bacteria **(A)** and fungi **(B)** at the genus level with fermentation products during aerobic exposure.

Figure 5 illustrated the relationships between the top 15 most abundant bacteria or fungi and fermentation products at the genus level. During fermentation 60 days and aerobic exposure 4 and 12 days, pH was negative correlated with *Lactobacillus* (*r* = –0.82, *p* < 0.001), *Issatchenkia* (*r* = –0.74, *p* < 0.01), and *Kazachstania* (*r* = –0.76, *p* < 0.01); WSC was positive with *Naumovozyma* (*r* = 0.73, *p* < 0.01) and *Issatchenkia* (*r* = 0.61, *p* < 0.05); lactic acid was positive with *Lactobacillus* (*r* = 0.58, *p* < 0.01)*;* AFB1 was negative with *Pantoea* (*r* = –0.69, *p* < 0.01), *Sporidiobolus* (*r* = –0.86, *p* < 0.05), and *Filobasidium* (*r* = –0.85, *p* < 0.001), and positive with *Bacillus* (*r* = 0.61, *p* < 0.05), *Wickerhamomyces* (*r* = 0.67, *p* < 0.01), and *Aspergillus* (*r* = 0.42, *p* < 0.01). The correlation analysis of DON was generally similar to AFB1.

## Discussion

The characteristics of raw materials, especially the DM and WSC contents, are important factors in determining the quality of silage fermentation. It is generally accepted that the optimal DM of silage is between 25.00 and 35.00%, while the theoretical WSC requirement for maintaining successful silage is above 50.00 g/kg DM (Wang et al., 2018; Li et al., 2019). In the present study, fresh WCWD had a higher DM content at 44.48%, and a relatively low WSC concentration of 34.30 g/kg DM. This illustrates the difficulty of obtaining high-quality silage by ensiling WCWD alone. These two factors for fresh AA were quite the opposite to WCWD, mixing these two materials for silage may result in a good quality feed, which is one of the objectives to carry out this study. The number of epiphytic LAB on fresh materials was above 5.50 lg cfu/g FM, which was high enough to convert WSC into organic acids and decrease pH under anaerobic condition, guarantee the effective fermentation (Oliveira et al., 2017). Moreover, bacillus, aerobic bacteria, and yeast, which are undesirable for silage, were more than 4.36 lg cfu/g FM in both two raw materials, might be a challenge to ensile directly without exogenous additives.

The pH of silage is an important parameter to indicate the degree of fermentation. A lower pH ensures better anaerobic fermentation and further inhibits the growth of harmful or undesirable microorganisms (Kung et al., 2018). In this research, through anaerobic fermentation for 3 days, the pH firstly declined to approximately 4.00 in LB and 20% AA, indicating that the WCWD ensiling was facilitated by adding LAB and 20% AA. Furthermore, at 2 days of aerobic exposure next to 60 days ensiling, pH values were both below 4.00 in LB and 20% AA, and it increased with the increase of AA in AA-treated groups, but it was still lower than that in WCWD. With the extension of aerobic exposure, pH in all groups significantly increased, while in 20% AA was still the lowest. This might be because after the fermented feed was opened and came into contact with the air, carbohydrate, organic acid, protein, and amino acid were decomposed by aerobic microorganisms, heat was generated and the pH increased (Kung et al., 2003). However, 20% AA treated to WCWD might exert an antibacterial effect continuously, slow down the proliferation of aerobic bacteria, and thus, prevent the rapid increase in pH.

Lactic acid, produced by LAB, leads to the reduction of pH during the early stage of ensiling. During the silage process in this research, the highest lactic acid concentration was observed in LB. The content of lactic acid in 20% AA was slightly higher compared with other groups; however, other proportions of AA didn’t exhibit corresponding effects. This might be explained by that LB and 20% AA inoculations accelerated lactic acid fermentation during ensiling. Similar results were also detected in whole-plant corn silage (Sun et al., 2021) and sugarcane top silage (Wang et al., 2020). With the extension of aerobic exposure time, lactic acid all decreased, but in 20% AA was highest among all groups at 12 days aerobic exposure. As lactic acid content could reduce the pH value of silage, effectively inhibit the growth of harmful bacteria and prevent the silage from spoilage, it might be one of the reasons for fermentation quality was good in 20% AA (Guo et al., 2018). The contents of acetic acid began to decrease among all groups from 15 days, this might be because pH did not change much or increased after that, and the acidity decreased, part of the acetic acid is combined with the salt group to form acetate (Guan et al., 2021). Contents of acetic acid in all AA-treated groups were significantly higher after ensiling 60 days. For appropriate acetic acid concentration that could inhibit yeast and improve aerobic stability, AA groups could significantly inhibit fungi and improve aerobic stability of silage, which were also consistent with the findings of Ma et al. (2022) and Wang et al. (2022b).

The DM content in 60 and 80% AA from 7 day and LB and 20% AA from 15 days of ensiling was increased. This result may be because the initial DM of LB and 20% AA group was 44.01 and 39.86%, which were not within the optimal DM range compared with 60% AA group (33.86%) and 80% AA group (33.22%), but the humid environment might promote the growth and reproduction of various bacteria to a certain extent. With the prolongation of fermentation time, LB and 20% AA showed a better inhibition effect on unwanted bacteria, especially aerobic bacteria and yeast, which reduced the loss of DM caused by metabolism and decomposition. Respiration by bacteria consumes some of the organic material during the fermentation, while releasing CO_2_ and H_2_O. This reduces the total amount of nutrients and decreases CP content, resulting in a “concentration effect” of the protein. The loss of CP in 20% AA was the least, especially at aerobic exposure time. This is probably because products such as short-chain peptides, free amino acids, and non-protein nitrogen hydrolyzed by CP can be used by some microorganisms to synthesize microbial proteins (He et al., 2020), increasing the true protein content. Proteolysis is one of the most important processes during silage, which is often hydrolyzed into non-protein nitrogen as NH_3_-N and peptide by microbial activity and protease (Wang et al., 2018). The ratio of NH_3_-N to total nitrogen reflects the degree of protein and amino acid decomposition during silage production, with a higher ratio indicating more protein decomposition and poor silage quality. It is generally accepted that if the NH_3_-N is less than 1/10 of the total nitrogen content, it indicates successful fermentation (Hassanat et al., 2007; Kung et al., 2018). In this study, NH_3_-N showed an uptrend with the prolonging of fermentation and aerobic exposure, which might be due to the presence of various microorganisms. The lowest growth rate has detected in 20% AA might be because lower pH inhibited the growth protein-hydrolyzing microorganisms, while highest growth rate has detected in 80% AA and AA, which was consistent with the higher pH value during aerobic exposure. The difference in the quality of roughage is reflected in the levels of NDF and ADF. In this study, the NDF and ADF contents in all groups have been declined after ensiling, suggesting fermentation breaks down cellulose to form volatile fatty acids, which are the main source of energy for ruminants (Ogunade et al., 2018).

Ensiling is a dynamic enzymatic and microbial reaction involving microorganisms such as LAB, bacillus, and yeast (Pahlow et al., 2003). The addition of LAB can accelerate the accumulation of lactic acid, lower pH values, and inhibit undesirable bacteria growth (Ni et al., 2017). In this study, from the beginning of the fermentation, the number of LAB was higher and the growth of undesirable microorganisms was lower in LB. On the 3 days of fermentation, numbers of bacillus and aerobic bacteria in LB were markedly lower, which might be due to the fast acidification is key to controlling the growth of harmful bacteria (Pahlow et al., 2003). Meanwhile, AA had an obviously inhibitory effect on harmful bacteria and fungi including *Aspergillus flavus*, *Escherichia coli*, *Colletotrichum fragariae*, and so on (Guan et al., 2019). The highest LAB numbers were observed in 20% AA from 15 days until the end of the aerobic exposure phase. This coincided with the fewest harmful microorganisms, lowest pH, and highest lactic acid content in 20% AA. During ensiling, the growth of yeast can consume large quantities of nutrients, increase pH and lead to the deterioration of silage (Li et al., 2021). In this experiment, all AA-treated groups inhibited yeast growth, while LB treatment proved ineffective. However, during aerobic exposure yeast proliferated rapidly in all groups, although increasing amounts of AA were able to slow down this deterioration, while 20% AA had the least counts of yeast at the end of the entire observation period. This finding confirmed previous observations that some yeasts can grow well in acidic and anaerobic environments and that lactic acid alone is insufficient to inhibit the growth of these fungi (Santos et al., 2016).

Silage can become contaminated with a variety of mycotoxins before harvest, during fermentation, and after the completion of silage. Ingestion of mycotoxin by ruminants can adversely affect their health and performance and pose a threat to food safety and human health. Furthermore, mycotoxins are extremely heat stable and cannot be adequately eliminated by conventional feed processing methods (Juan et al., 2020). Fusarium head blight (FHB) is one of the most important diseases that occur during wheat cultivation. It is widespread and affects a large area, producing a variety of toxic metabolites, causing a reduction in wheat yield and quality (Abbas and Ylimattila, 2022). Agustina et al. (2017) identified several mycotoxins in wheat silage, with AFB1 and DON being the most commonly encountered and abundant. Currently, strategies aiming to degrade these contaminating toxins by the addition of microorganisms or Chinese herbal medicines are being explored. Converting mycotoxins into non-toxic metabolites by enzymes produced by living microorganisms or the direct addition of enzymes present in plants holds great promise due to the high efficiency, specificity, and environmentally friendly nature of these approaches (Gallo et al., 2021). The addition of heterofermentative LAB is able to shift silage fermentation toward heterolactic pathway and inhibits the growth of toxigenic fungi. As a consequence, it could delay the onset of aerobic deterioration after exposure to air of the silage, and stave off mycotoxin synthesis (Ferrero et al., 2019; Mugabe et al., 2019). In the present experiment, toxin contents significantly increased compared with fresh materials after 60 days ensiling; while AFB1 in 20% AA had the lowest content. This may be due to the fact that the aerobic mold–producing mycotoxins is still active during the early aerobic respiration stage (0–3 days) after fermentation; although pH further decreased, LAB became the dominant flora, and mold activity was limited and toxin production gradually decreased, it still continued to accumulate (Aragon et al., 2011; Cheli et al., 2013). According to the results, the addition of AA can effectively inhibit mycotoxin production. It presented the lowest AFB1 level in 20% AA during aerobic exposure. Meanwhile, DON contents in all groups still increased, but 20% AA had the lowest DON level throughout the aerobic exposure. Mycotoxins are closely related to the structural and quantitative changes of fungi community, and AA could control toxin contents by inhibiting fungal growth and reducing the abundance of fungi (Wang et al., 2022a), which is consistent with the results of this experiment.

Feed quality is drastically influenced by the species composition and abundance of dominant microorganisms during the fermentation process (Hassanat et al., 2007). After 60 days ensiling, the richness of bacteria and fungi in LB and 20% AA groups were lower compared with other groups, which may because of the relatively lower pH values (3.85 and 4.11). McAllister et al. (2018) also reported that inhibiting the reproduction of unfavorable microorganisms in silage would reduce the lower abundance of the microbial composition, thus improving the silage quality. During aerobic exposure, the microbial richness in WCWD decreased dramatically. Similar observations were also reported by Zhang et al. (2019) and Wang et al. (2022a). At 2 and 4 days, the number of microorganisms in LB, 20% AA and AA groups were relatively high, and the compositions of microbial community in LB and 20% AA were gradually similar. This might because microbialcommunities in silage treated with LAB and AA changed along with environment from anaerobic to aerobic (Wang et al., 2022b).

Changes in the main microorganisms during fermentation and aerobic exposure are closely related to the quality of the feed. In this study, after 60 days ensiling, the predominantly bacterial shifted from Proteobacteria to Firmicutes. Firmicutes is a vital acid hydrolytic microbes under anaerobic condition that including the majority of bacteria involved in lactic acid fermentation. It could influence metabolism and maintain the homeostasis of the internal environment through their specific flora structure, activity, and metabolites (Preston et al., 2005). This phenomenon that the dominance of Firmicutes after ensiling might be because of the anaerobic conditions, which were beneficial to the growth of Firmicutes. The relative abundance of Proteobacteria increased as the extension of aerobic exposure, and Firmicutes was gradually replaced by Proteobacteria, especially significantly in WCWD group. These results are in accordance with the report of Ali et al. (2020), it might be because an increased in pH and decreased in organic acids are not beneficial to the proliferation of Firmicutes. Dynamic changes in the bacterial community from Gram-negative to Gram-positive microbes during ensiling indicate that fermentation could inhibit the proliferation of pathogenic bacteria widely presented in raw materials, and AA played same and even better roles than LAB. Meanwhile, aerobic exposure made a comeback or even exacerbation of harmful bacteria, AA and LAB slowed down the aerobic deterioration of WCWD to a certain extent, but they cannot be fundamentally avoided. Therefore, it should be fed as soon as possible after opening.

*Pantoea* represents the most dominant genera in fresh materials (Ma et al., 2022), and the WCWD in this study was no exception. However, the relative abundance of *Pantoea* decreased during the ensiling and the following aerobic exposure, while it was higher at 20% AA, this is consistent with Wang et al. (2021). *Pantoea* has the ability to reduce NH_3_-N content and pH value of silage, this may be the reason for lower pH values and NH3-N contents in 20% AA. Moreover, the negative correlation between AFB1 and *Pantoea* was observed, which was in line with the aforementioned lower AFB1 content in 20% AA during fermentation and aerobic exposure. At the early stage of ensiling, *Lactobacillus* usually dominates and converts plant carbohydrate into organic acid to decrease pH of silage after some plant cell and aerobic microorganisms consume oxygen. It can inhibit the growth of *Enterobacteria* and other undesirable microorganisms such as yeast and mold (Sun et al., 2021). In the present study, after 60 days ensiling, *Lactobacillus* abundance increased in all groups, especially in 20% AA. Once the anaerobic environment is destroyed, *Lactobacillus* numbers decreased with extended periods of aerobic exposure, while their abundance was relatively better preserved in 20% AA. *Lactobacillus* was positively correlated with lactic acid and negatively with pH during ensiling and aerobic exposure in the study. Those abovementioned suggested that the satisfactory fermentation quality of silage in 20% AA might be higher abundance *Lactobacillus* dominating the fermentation process. *Enterobacter* is generally considered to be undesirable during fermentation because it can ferment WSC and lactic acid to ethanol, 2,3-butanediol or endotoxins, which may cause degradation of fermentation quality and feed contamination (McGarvey et al., 2013). This is also confirmed in the present study. *Enterobacter* was positively correlated with NH_3_-N and AFB1 during the whole experiment, and it was rarely detected in LB and 20% AA treated groups after aerobic exposure, this may also be one of the reasons for fermentation quality remained well in these 2 groups. With the extension of aerobic exposure, *Acinetobacter* increased, and the abundance in 20% AA was the lowest, while that in WCWD was the highest. *Acinetobacter* proliferates rapidly under aerobic conditions, causing the deterioration of silage, and posing a risk to animals and humans (Liu et al., 2019). Correlation analysis showed that *Acinetobacter* and AFB1 had a positive correlation also proved this point.

In the present study, fungi were represented by Basidiomycota and Ascomycota. Basidiomycota is a major lineage of fungi containing more than 40,000 species. Basidiomycota represents almost a third of all known fungal species including mushrooms, plant pathogenetic smut, rust, and industrially important forms of yeast. These play an essential role in the ecosystem and participate in the recycling of nutrients (He and Zhao, 2021). Ascomycota, comprised of approximately 11,000 species, is also a diverse and species-rich phylum in the kingdom of fungi. It contains a broad range of life modes including pathogenic, saprobic, and endophytic (Ali et al., 2020). Basidiomycota was the dominant phylum in fresh materials, and the abundance of Ascomycota significantly increased and occupied the dominant position among all groups through the ensiling process and aerobic exposure time. This result was in accordance with the findings of Liu et al. (2019) and Zhang et al. (2019). Interestingly, there were no notable differences in the abundance of Basidiomycota and Ascomycota between WCWD, LB, and 20% AA silages, suggesting that fungal composition at the phylum level was rarely affected by the presence of inoculants or additives during fermentation and aerobic exposure.

Members of Sporomiaceae occur as saprobes on a wide range of substrates, including herbivore dung, plant debris, wood, and soil (Phukhamsakda et al., 2016). They were found to be relatively abundant in fresh WCWD. In contrast, *Filobasidium* took a dominant position in AA, which is widely found in most traditional medicinal plants and is beneficial to their host plant (Chen et al., 2020). The amounts of AFB1 and DON showed a markedly negative correlation with *Filobasidium*, and lower levels of toxins in fresh AA might be attributed to this. Species of the genera *Aspergillus*, *Fusarium*, *Penicillium*, and *Monascus* have been frequently reported, many of them able to produce mycotoxins, which mainly are AFB1 and DON (Cheli et al., 2013). Untreated WCWD had the highest contents of *Aspergillus* and *Monascus*, while 20% AA had the least both during fermentation and aerobic exposure. This is consistent with the positive correlation between the abundance of *Aspergillus* and *Monascus* with AFB1 and DON contents, respectively. After a longer period of aerobic conditions, Wickerhamomyces became remarkably abundant and started to outnumber *Aspergillus* and *Monascus* in 20% AA and AA. Several studies demonstrated that Wickerhamomyces could inhibit the production of mycotoxins, while enhancing the richness and complexity of volatile aroma compounds (Liu et al., 2021; Raynaldo et al., 2021). In the present study, there was no significant correlation between AFB1 and Wickerhamomyces. Similar findings were reported by others, corroborating this observation (Deepa et al., 2019; Restuccia et al., 2020). These results clearly showed that AA can inhibit the growth of undesirable microorganisms, thereby improving the quality of fermented feed both during fermentation and following aerobic exposure. These findings provide further evidence that AA can used as useful additives in the production of fermented animal feed.

## Conclusion

This study evaluated the fermentation quality, chemical composition, microbial population, and mycotoxin of silage ensiling (1, 3, 7, 15, 30, and 60 days) and aerobic exposure (2, 4, and 12 days) treated by LB and AA. Compared with treated by LB, throughout the ensiling and exposure, 20% AA could improve the quality of WCWD by higher contents of lactic acid, lower NH_3_-N, enhance *Lactobacillus* abundance. Meanwhile, 20% AA reduced *Enterobacter*, *Acinetobacter*, and *Aspergillus*, effectively degraded AFB1 and DON. To sum up, addition of 20% AA to WCWD could more effectively increase the abundance of LAB, reduce the production of harmful microorganism and mycotoxins, and improve the quality of silage, especially in aerobic exposure stage. The results of this research provide a preliminary reference toward establishing the possibility that AA might be used as efficient feed additive.

## Data availability statement

The data presented in this study are deposited in the https://www.ncbi.nlm.nih.gov/, accession number: PRJNA894537.

## Author contributions

ZW, ZT, and HP contributed to the conception and design of the study. GW organized the data curation. LW performed the statistical analysis. ZW and HP wrote the first draft of the manuscript. GQ and YW were in charge of project administration. All authors read and agreed to the published version of the manuscript.

## Footnotes

1
https://login.majorbio.com
